# Graph Learning for Fake Review Detection

**DOI:** 10.3389/frai.2022.922589

**Published:** 2022-06-20

**Authors:** Shuo Yu, Jing Ren, Shihao Li, Mehdi Naseriparsa, Feng Xia

**Affiliations:** ^1^School of Software, Dalian University of Technology, Dalian, China; ^2^Institute of Innovation, Science and Sustainability, Federation University Australia, Ballarat, VIC, Australia; ^3^Global Professional School, Federation University Australia, Ballarat, VIC, Australia

**Keywords:** graph learning, fake review detection, anomaly detection, social computing, data science

## Abstract

Fake reviews have become prevalent on various social networks such as e-commerce and social media platforms. As fake reviews cause a heavily negative influence on the public, timely detection and response are of great significance. To this end, effective fake review detection has become an emerging research area that attracts increasing attention from various disciplines like network science, computational social science, and data science. An important line of research in fake review detection is to utilize graph learning methods, which incorporate both the attribute features of reviews and their relationships into the detection process. To further compare these graph learning methods in this paper, we conduct a detailed survey on fake review detection. The survey presents a comprehensive taxonomy and covers advancements in three high-level categories, including fake review detection, fake reviewer detection, and fake review analysis. Different kinds of fake reviews and their corresponding examples are also summarized. Furthermore, we discuss the graph learning methods, including supervised and unsupervised learning approaches for fake review detection. Specifically, we outline the unsupervised learning approach that includes generation-based and contrast-based methods, respectively. In view of the existing problems in the current methods and data, we further discuss some challenges and open issues in this field, including the imperfect data, explainability, model efficiency, and lightweight models.

## 1. Introduction

**Figure 1:**
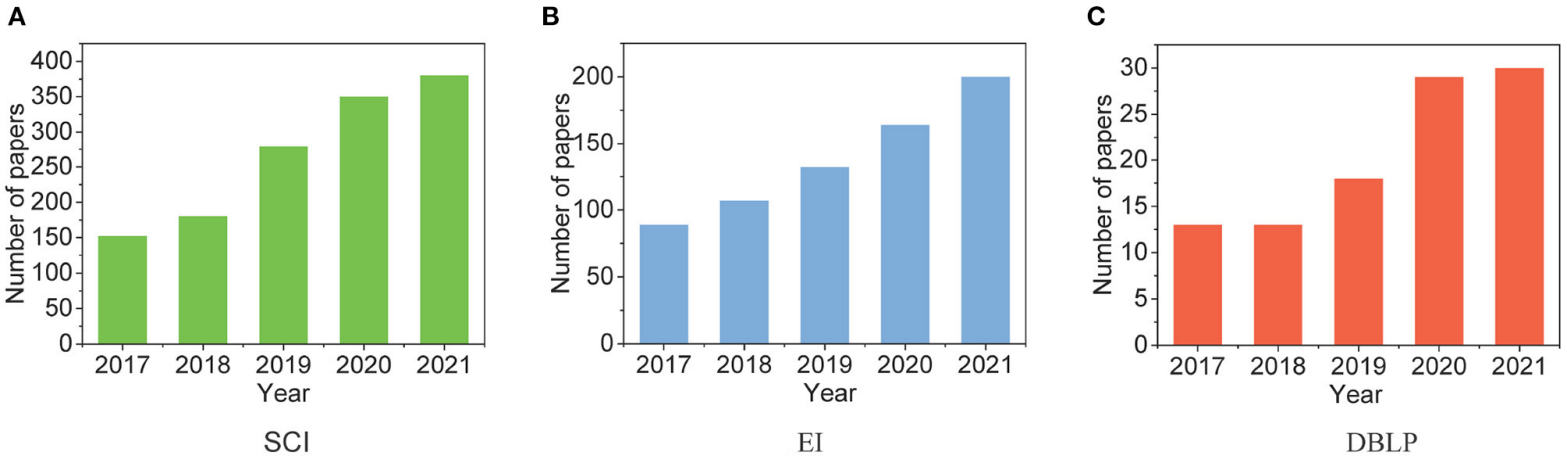
Changes in the number of papers related to fake reviews in the past 5 years. **(A)** SCI. **(B)** EI. **(C)** DBLP.

With the prosperity of web services and social networks, e-commerce nowadays has gained tremendous popularity among a wide range of people. When this prosperity significantly boosts the sales of online businesses, it also leads to a considerable number of malicious sellers and spam activities on business websites. Driven by the monetary incentives, they utilize the vulnerability of online websites to make more profits, such as using fake identities in online review systems; thus, manipulating the reputation of the products and brands (Li et al., 2021a; Shehnepoor et al., 2021; Wang L. et al., 2021; Yang et al., 2021). Research on fake reviews have grown noticeably in the last 5 years, and Figure 1 presents the research status by showing the number of papers with keyword “fake reviews” on SCI, EI, and DBLP, respectively. The anonymity characteristic of the Internet makes it challenging for the websites to handle fake reviews, and the high volume of freelancers and botnets worsen this situation (Wang et al., 2019; Wen et al., 2020; Hou et al., 2021; Li et al., 2021b). Moreover, fake reviewers adopt camouflage techniques to hide their identities (Hooi et al., 2016). These put forward the challenging problem of fake review detection.

Previous studies focus on distinguishing the content of news and learning text or image features to investigate fake review detection (Yuan et al., 2019; Branco et al., 2020). The rich information on the social context of reviews is then extracted and further analyzed (Yuan et al., 2017). Though context-based methods have made some progress, the explicit and implicit correlations among users and review contents are still unexplored. The effectiveness and accuracy of fake review detection are thus limited. As a result, graphs are built to represent interactive and complex relationships in fake review detection tasks. These methods utilize graph-structured data to formulate a binary relationship between reviews and reviewers, and they achieve significant performance in fake review detection. Despite the progress in the current studies, fake review detection still faces several challenges. Many models only focus on pairwise relationships and ignore the higher-order relationships. Moreover, though bipartite graphs are constructed, the representation of multi-source data is still unexplored.

Meanwhile, Graph Learning refers to the applications of machine learning models on graph data. With the rapid development in recent decades, graph learning has proven to be of great significance because of its wide applications. Graph learning-based fake review detection has thus been proposed and studied extensively in recent years. There are the following benefits of graph learning-based fake review detection. Firstly, most data in fake review detection contain rich relationships with each other; thus, the graphs effectively leverage the inter-connectivity in these real-world data (Yuan et al., 2017; Yu et al., 2017; Ma et al., 2022). Graphs powerfully capture the correlations among inter-dependent data objects. This nature is even more obvious in online review systems where users, items, attributes, and context are tightly associated with and impact each other by relations (Jerripothula et al., 2020; Rossi et al., 2020; Wang et al., 2020; Liu et al., 2021d). A variety of graphs are generated from data in review systems, and they significantly improve the performance of fake review detection. Second, graph learning effectively learns complicated relations and extracts knowledge from different kinds of graphs (Xu et al., 2020; Wang W. et al., 2021; Xia et al., 2021a). The objective of graph learning is to extract required features from graphs; then, the graph representation is applied for specific tasks (Guo et al., 2021; Xia and Ku, 2021; Xia et al., 2021b; Liu J. et al., 2022; Wang, 2022). In detail, many graph learning techniques, such as graph neural networks (GNNs), have been developed to learn the specific type of relations in the graph models and have been proven effective. Therefore, it is sensible to employ graph learning to model various relations in online review systems.

This paper presents the first literature survey of graph learning techniques for fake review detection. Though there have been surveys and reviews about graph-based anomaly detection (Akoglu et al., 2015; Pourhabibi et al., 2020) and deep learning-based graph anomaly detection (Ma et al., 2021), none of them focus on anomaly detection's down-stream application-fake review detection. While, Istanto et al. (2020) reviewed the fake review detection techniques published between 2015 and 2019, our survey focuses on graph learning's applications in one specific area of anomaly detection. Furthermore, our work focuses on techniques with graph learning and covers a wide range of time.

### 1.1. Contributions

The main contributions of this paper are summarized below:

- We provide a comprehensive analysis of the key challenges in graph learning-based fake review detection to assist readers with a better understanding of this downstream task.
- We summarize the current research progress in graph learning-based fake review detection, including supervised and unsupervised methods.
- We share and discuss significant future directions of graph learning-based fake review detection by summarizing open issues and challenges.

The rest of this paper is organized as follows: Section 2 explains the categories of fake reviews and presents a comprehensive study of the recent literature on the fake review issue. Section 3 reviews the graph learning methods for fake review detection. Section 4 summarizes the benchmark datasets used in fake review detection task. Section 5 analyzes the open issues and possible future directions of fake review research. Finally, Section 6 concludes this paper.

## 2. The Study of Fake Reviews

In this section, we first illustrate the typical examples of fake reviews as well as the existing categories for fake reviews. Then, we present a comprehensive study on the fake review issue and discuss our taxonomy for fake review detection approaches.

### 2.1. Categories of Fake Reviews

According to previous studies (Jindal and Liu, 2008; Li A. et al., 2019) and our summary, we categorize the fake reviews based on two factors: (a) their content and (b) their purposes. Definitions and examples of fake reviews are given in Table 1.

**Table 1:** Examples of fake reviews.

**Types of fake review**	**Definition**	**Example**
Untruthful opinions	These reviews intentionally misguide users of the review system by unjustly reviewing and rating target objects to manipulate the products' reputation.	(1) This little place in Soho is wonderful. World-class service. (2) Their artichoke chicken salad is the worst in NY.
Exclusive reviews	These reviews are given exclusively to specific brands, manufacturers, or sellers.	(1) The food is amazing! My friends and me are definitely coming back to this place. (2) Delicious, consistent, well-priced. Feels like its made with love.
Non-reviews	Non-reviews include two main sub-streams: (1) Advertisements and (2) Irrelevant content without opinions.	(1) Register to receive a gift. (2) akhdbfl (garbled)
Duplicates reviews	Different accounts post duplicate or near-duplicate reviews on products, either the same or different.	(1) Really charming. It is a great place to have a low-key lunch. (2) The food is simple and effective you should go. (3) It is a great place to have a low-key lunch. (4) The food is simple and effective- you should go.

#### 2.1.1. Untruthful Opinions

These reviews intentionally misguide the system users by unjustly reviewing and rating target objects to manipulate the products' reputation. The ratings are extremely high or low, and the review contents are either high praise or unfavorable comments.

#### 2.1.2. Exclusive Reviews

These reviews target specific brands, manufacturers, or sellers. They are considered fake reviews whether they seem useful or not. That is because they are biased toward their targets and not objective enough.

#### 2.1.3. Non-reviews

Non-reviews include two main sub-streams: (1) advertisements and (2) irrelevant contents without opinions (e.g., questions, answers, and random texts).

#### 2.1.4. Duplicates Reviews

These are clearly spam. For instance, different accounts post duplicate or near-duplicate reviews on products, either the same or different.

### 2.2. A Taxonomy of Fake Review Detection Approaches

**Figure 2:**
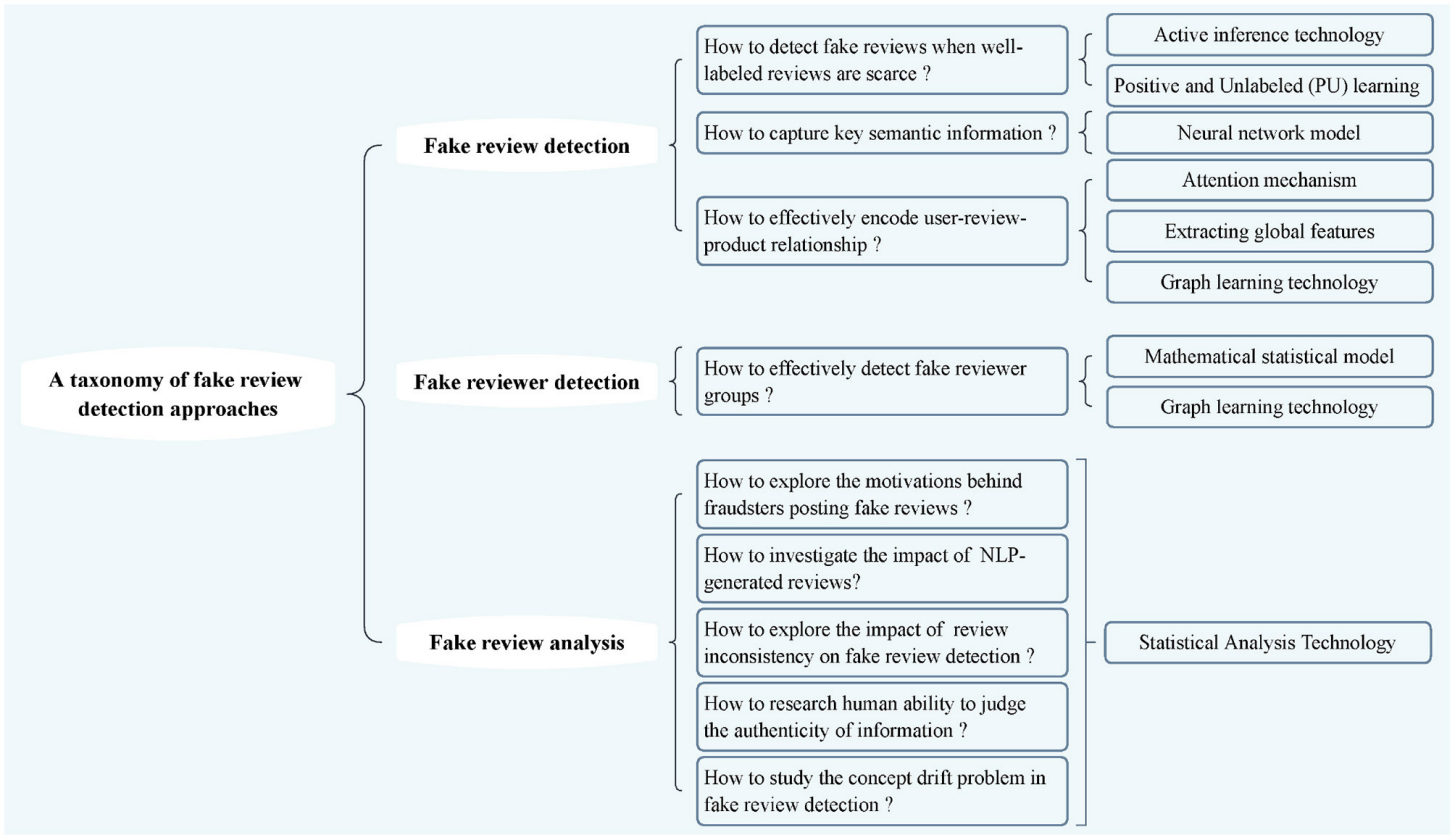
A taxonomy of fake review detection approaches.

As illustrated in Figure 2, we summarize the fake reviews into three types, including fake review detection, fake reviewer detection, and fake review analysis. Figure 2 summarizes comprehensive research around the taxonomy of fake review detection approaches. We will introduce these approaches in detail next.

#### 2.2.1. Fake Review Detection

Online reviews play a pivotal role in consumers' decision-making. However, the existence of some fake reviews seriously misleads consumers' choices of products. Therefore, a lot of works are devoted to studying effective fake review detection. However, due to the high cost of manual data tagging, well-labeled reviews are very scarce. To address this problem, Rayana and Akoglu (2016) propose a collective opinion spam detection framework, which adopts active inference technology to select valuable nodes for labeling. They mainly design a label selection strategy based on three key characteristics of valuable nodes. They judge the node's value within a small budget so that the node is labeled and utilized for fake review detection. He et al. (2020) leverage Positive and Unlabeled (PU) learning to detect fake reviews, i.e., only a small number of positive samples and a large number of unlabeled samples are used to classify reviews. This scheme avoids the reliance on manually labeled data. Furthermore, they combine user behavior density to analyze fake reviews, which improves detection accuracy.

In addition, some studies are concerned about how to extract the useful features of reviews to detect fake reviews more accurately. Ren and Zhang (2016) design a neural network model which extracts document features to obtain corresponding representations. Compared with the manual discrete features model, the learn document vectors captured more critical semantic information, which leads to improving the performance of fake review detection. Fahfouh et al. (2020) exploit Paragraph Vector Distributed Bag of Words (PV-DBOW) and the Denoising Autoencoder (DAE) to obtain a global representation of a review. They focus on semantic information in the context of reviews to break the limitations of traditional classifiers. Hajek et al. (2020) explore the importance of hidden emotions contained in review texts. The proposed neural network model analyzes the semantic information in reviews. Also, the model paid attention to emotional features expressed by consumers in reviews when learning review embeddings. However, the above methods only consider the semantic features of the review and ignore its connection with the user and the product.

To address the problem mentioned above, Wang et al. (2016) learn the representation of reviews in a data-driven manner to avoid relying on experts' knowledge. Meanwhile, they combine the reviewer, product, and review text features to learn the representation of the review, which makes full use of global information and improves the performance of fake review detection. Yuan et al. (2019) design a hierarchical fusion attention network (HFAN) to learn the representation of reviews. It first extracts the semantic features of users and products. Then, it generates corresponding representations and encodes the user-review-product relationship to get the final review representation. This work highlights the importance of user and product information for learning review representation. Yu et al. (2019) mainly analyze the behavior of the stakeholders of the reviews and judge the falsity of the reviews. Specifically, they propose three indicators to calculate the fake degree of individuals, groups, and merchants. Then, they integrate them to detect fake reviews. Budhi et al. (2021) combine content and behavior features to detect fake reviews. To obtain global information, they summarize 133 features, including review text, user behavior, and product behavior features, respectively. Meanwhile, they also designed two sampling methods to solve the negative impact of imbalanced datasets on fake review detection.

The above methods have proved that the features of reviews, users, products, and their relationships all play a pivotal role in detecting fake reviews. The construction of the review graph effectively assists us to learn this information. Therefore some researchers applied graph learning for fake review detection, which leads to satisfying results. For instance, Li A. et al. (2019) construct a heterogeneous graph (i.e., Xianyu Graph) and a homogeneous graph (i.e., Comment Graph) to learn the local and global contexts of a review, respectively. Furthermore, they utilized a GCN-based Anti-Spam (GAS) model to detect fake reviews in Xianyu. Sun and Loparo (2019) employ all heterogeneous data in social networks to detect fake reviews and convert them into classification tasks on heterogeneous information networks. Moreover, Noekhah et al. (2020) present a novel heterogeneous graph (MGSD) model to capture the relationships among entities, and their corresponding weight. They combine multiple features to obtain a new set of features for fake review detection. Various studies have confirmed the effectiveness of the graph learning method for fake review detection.

#### 2.2.2. Fake Reviewer Detection

Driven by lots of profit, some businesses or users are devoted to publishing fake reviews to influence the consumption behavior of the consumers. Such behavior often leads to damaging the trust between businesses and consumers. Therefore, fake reviewer detection has attracted the increasing attention of researchers. For example, Li H. et al. (2017) analyze the number of reviews made by reviewers over a time period and found that they follow a fixed pattern. Specifically, multiple fake reviewers are likely to actively review the same set of products in a short time period (i.e., co-bursting). Thus, they design a two-mode Labeled Hidden Markov Model (LHMM) to detect fake reviewers. Kaghazgaran et al. (2018) utilize the Two-Face system to detect review manipulators. The system identifies the users with similarities to seed users through a so-called suspicious graph. They find that the difference in behavior features of manipulators and regular users is relatively small. Therefore, review manipulators are easier to identify by comparing their social features with regular users. Dhawan et al. (2019) believe that it would cause more dire consequences if fake reviewers collectively post fake reviews; thus, they propose a new framework to detect fake reviewer groups. Besides, Byun et al. (2021) construct a user similarity projection graph and divide the corresponding community. In the next step, they extract the abnormal feature to classify opinion spammers. Xu et al. (2021) firstly constructs the reviewer-projection graph; then, they adopt the Clique Percolation Method (CPM) to detect the opinion spammer group. The above methods demonstrate their superior performance in detecting fake reviewers.

#### 2.2.3. Fake Review Analysis

There have been many studies on fake review analysis to deal with the exponential growth in the number of fake reviews. Luca and Zervas (2016) analyzes fake reviews on Yelp to explore the primary motivation behind the fake reviewers who post fake reviews. For example, they find that chain restaurants are less likely to receive fake reviews when compared to independent restaurants due to their established reputation. This finding helps people understand how a business's reputation affects its motivation for posting fake reviews. Hovy (2016) explores the impact of the application of natural language processing techniques in the generation of fake reviews. They utilize various language models to generate fake reviews based on meta-information and try to detect these fake reviews. Finally, they find that NLP-generated reviews are more difficult to detect; thus, they reflect the dual character of the application of NLP techniques.

In addition, Shan et al. (2021) explore the impact of review inconsistency on fake review detection. They present three types of review inconsistency, including rating-sentiment inconsistency, content inconsistency, and language inconsistency. Based on their findings, the review inconsistency of fake reviews is noticeably high. Therefore, review inconsistency is a fruitful measure to improve the accuracy of fake review detection. Banerjee and Chua (2021) are dedicated to researching users' perception of language nuances. Thus, they invite 380 participants to judge the authenticity of three hotel reviews. The results verify that linguistic cues assist the users in judging the authenticity of reviews to a certain extent. However, the human ability to judge the authenticity of information is almost equivalent to random guessing. Mohawesh et al. (2021) focus on the drift problem concept in fake review detection. They find that the drift problem concept is common in fake review detection and the classifier performance decreases over time which reminds us to update the classifier frequently. Multiple studies on fake review analysis from different perspectives bring us comprehensive thinking and provide new ideas for problem-solving.

## 3. Graph Learning for Fake Review Detection

Graph Neural Networks (GNNs) have accomplished decent success in many tasks (e.g., node classification, sub-graph classification, graph classification, link prediction) owing to their capability of capturing node attributes and graph structure information. Therefore, many fake review detection methods thoroughly investigate GNNs to utilize their powerful capacities. It should be noted that Section 2 mainly introduces the definitions of different kinds of fake reviews and relevant fake review detection tasks, while this section focuses on graph learning-based methods. Instead of solving one task with one approach, graph learning-based models have their advantages in coping with multiple tasks at the same time.

In this section, GNN-based methods for fake review detection are classified into two categories: (a) supervised and (b) unsupervised. The supervised methods consider fake review detection a binary classification problem, while the unsupervised methods define it as a cluster problem. Here, we firstly summarize the supervised methods; then, we follow our discussion by explaining the unsupervised methods. Table 2 summarizes the important notations that are used in this paper. Table 3 summarizes the main characteristics of the graph learning papers for fake review detection. We category the methods to three detection tasks as listed in Section 2.2, wherein, “FRD”, “FRerD”, and “FRA” represent “Fake Review Detection”, “Fake Reviewer Detection”, and “Fake review Analysis”, respectively. In Table 3, we list several representative graph learning methods for fake review detection. These methods are specifically compared in several perspectives. GAS, PC-GNN, IHGAT, AO-GNN are supervised methods, while DeepFD, IN-GNN, and PAMFUL are unsupervised. Wherein, IHGAT focuses on link level and represents relation, others focus on node level. PAMFUL can only detect fake reviewer while other methods except PC-GNN can detect fake review. PC-GNN can recognize both fake review and fake reviewer at the same time. Among these methods, PC-GNN, AO-GNN, and DeepFD are with better generalization ability (Du et al., 2020; Betlei et al., 2021; Hibshman et al., 2021). Some detailed parameter settings are unspecified and some datasets are not publicly available such as Xianyu Graph, Alibaba Review Graph, and Alibaba Group, thus limiting the repetition of these methods to some extent.

**Table 2:** Commonly used notations with explanations.

**Notation**	**Explanation**
G	A graph.
V	The set of nodes in a graph.
E	The set of edges in a graph.
**X**	Node feature matrix of a graph
*v*_*i*_	A node in the node set V
*e*_*i,j*_	An edge in the edge set E
**h**_*i*_	The node representation vector of node vi.
C	Unlabeled node set.
**Z**	Output representation of the encoder.
**A**	The adjacency matrix of a graph.
$\hat{\text{A}}$	The reconstruction adjacency matrix.
$\hat{\text{Z}}$	The reconstruction feature matrix.
**D**	The node degree matrix.
σ(·)	Activation function

**Table 3:** Comparative review of graph learning methods for fake review detection.

**Methods**	**Detection task**	**Task level**	**Supervised/unsupervised**	**Scalability**	**Generalization ability**	**Datasets**
GAS (Li A. et al., 2019)	FRD	Node	Supervised	✓		Xianyu Graph
PC-GNN (Liu et al., 2021b)	FRD& FRerD	Node	Supervised	✓	✓	YelpChi, Amazon,
						and Alibaba Review Graph
IHGAT (Liu et al., 2021a)	FRD	Link	Supervised	✓		Alibaba Group
AO-GNN (Huang et al., 2022)	FRD	Node	Supervised	✓	✓	YelpChi, Amazon, and Books
DeepFD (Ding et al., 2019)	FRD	Node	Unsupervised	✓	✓	Yelp, Amazon, and DDos
IN-GNN (Liu B. et al., 2022)	FRD	Node	Unsupervised	✓		MisInfdect and Pheme
PAMFUL (Zhao et al., 2022)	FRerD	Node	Unsupervised			Bitcoin-Alpha, Weibo

### 3.1. Supervised Methods

**Figure 3:**
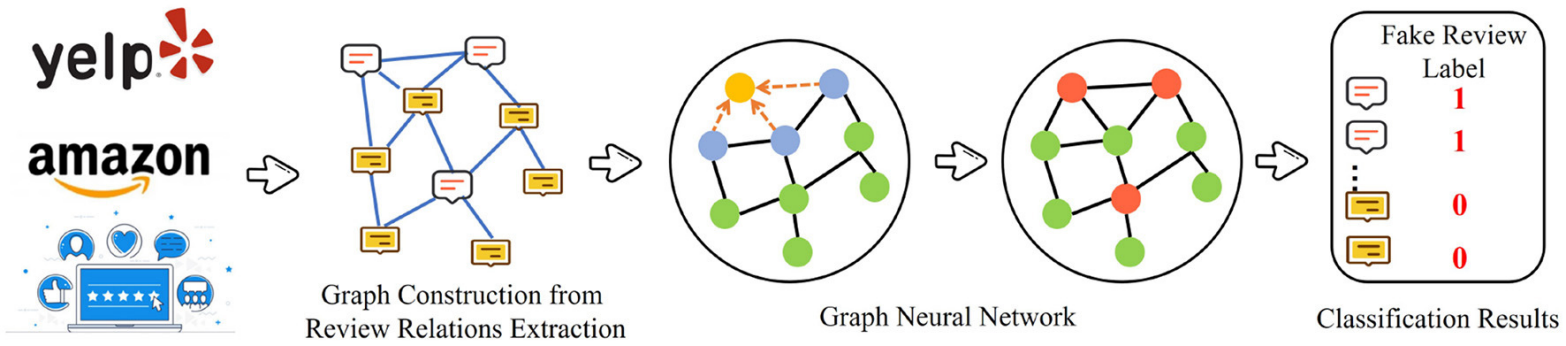
Supervised methods with Graph Neural Network for fake review detection.

Fake review detection is defined as a task that determines whether a review is fake or not. Therefore, some studies consider it as a binary classification task, which can be defined as G={V,E,C}, where each node in V has been labeled either fake or not in C. The supervised methods identify the fake nodes that significantly differ from the normal nodes in G. Figure 3 illustrates a general framework of the supervised methods with graph neural networks for fake review detection. GraphSAGE, proposed by Hamilton et al. (2017) and GCN proposed by Kipf and Welling (2017) are commonly used comment node embedding method. Li A. et al. (2019) adopt Graph Convolutional Networks (GCNs) to address the anti-spam problem at Xianyu (The largest second-hand goods app in China). Their method is called GCN-based Anti-Spam (GAS), which contains two primary inputs: (a) XianYu Graph (heterogeneous graph) and (b) Comment Graph (isomorphic graphs). The GCN-based methods mainly focus on isomorphic graphs. The graph which is utilized in the fake review detection process contains two types of nodes: (a) user and (b) item. Traditional GCN can not be processed directly; however, GAS extends GCNs for heterogeneous graphs to obtain local information and aggregate the information to the edges from the Xianyu Graph. The formula of each layer in GAS is presented as follows:


(1) $$\begin{array}{l} {\text{h}}_{e}^{l}=\sigma \left({\text{W}}_{E}^{l}\cdot AGG_{E}^{l}\left({\text{h}}_{e}^{l-1},{\text{h}}_{U\left(e\right)}^{l-1},{\text{h}}_{I\left(e\right)}^{l-1}\right)\right) \end{array}$$

wherein, ${\text{h}}_{e}^{l}$ is the representation of the edge and ${\text{W}}_{E}^{l}$ is the learning parameter, *AGG* is the concatenation operation. ${\text{h}}_{e}^{\text{h}-1}$, ${\text{h}}_{U\left(e\right)}^{l-1}$, and ${\text{h}}_{I\left(e\right)}^{l-1}$ are the edge, user, and item embeddings from the *l* − 1 layer. In addition, GAS considers global information through the Comment Graph. The node in the Comment Graph represents the comment, while the edge conveys the similarity between the comments. Moreover, GCN is utilized to obtain global information. Finally, local and global information are spliced and classified through labeled data sets.

Liu et al. (2021b) propose a method called PCGNN for imbalanced supervised learning. The imbalanced supervised learning proves to be a suitable approach for fake review detection. In this approach, they develop a label-balanced sampler to pick nodes and edges for sub-graph training. Then, they design a neighborhood sampler to choose neighbors for over-sampling the minority class and under-sampling the majority class neighborhoods, respectively. The sampler picks nodes and edges for the construction of the adjacent matrix. In the chosen step, the sampler generates samples for the minority class and under-samples the neighbors in the majority class. The overall loss function is formulated as follows:


(2) $$\begin{array}{l} L=L_{gnn}+\alpha L_{dist}, \end{array}$$

wherein, $L_{gnn}$ is the cross-entropy loss of the graph neural network. $L_{dist}$ denotes the loss for learning the parameter in the neighborhood sampler. α denotes the balanced parameter.

The majority of recent studies have put their efforts into the innovation of the new representation method; however, a few works focus on fake review detection. With the rapid emergence of fake reviews, we encourage more studies on fake review detection in a supervised way.

### 3.2. Unsupervised Methods

In some circumstances, fake review detection is defined as an unsupervised problem. For example, when background knowledge is not available to mark the data, unsupervised fake review detection methods effectively identify the fake reviews. This problem is defined as a graph G={A,E}, where the unsupervised methods learn a mapping function to embed the node features to a latent space. Berahmand et al. (2021) propose a new random walk model to integrate network structure and node attributes, based on the assumption that two nodes on the network will be linked since they are nearby in the network, or connected for the reason of similar attributes. The unsupervised methods detect all fake review nodes in the graph automatically. The detection of fake review nodes is based on the Poisson distribution or fake score of the nodes in a low-dimensional latent space. There are two groups of unsupervised fake review detection methods: (a) generation-based and (b) contrast-based methods, respectively. This categorization takes the different designs of pretext decoders and objective functions into account.

The generation-based methods focus on retaining more structural information by reconstructing the input graph, and they minimize the differences between the reconstructed and the input graphs, respectively. However, the contrast-based methods maximize the difference between the two corresponding views. As for the fake review detection, the generation-based methods pay more attention to the detection step after encoding, while the contrast-based methods pay more attention to the design of the discriminator; thus, they directly detect the fake review node.

#### 3.2.1. Generation-Based Methods

**Figure 4:**
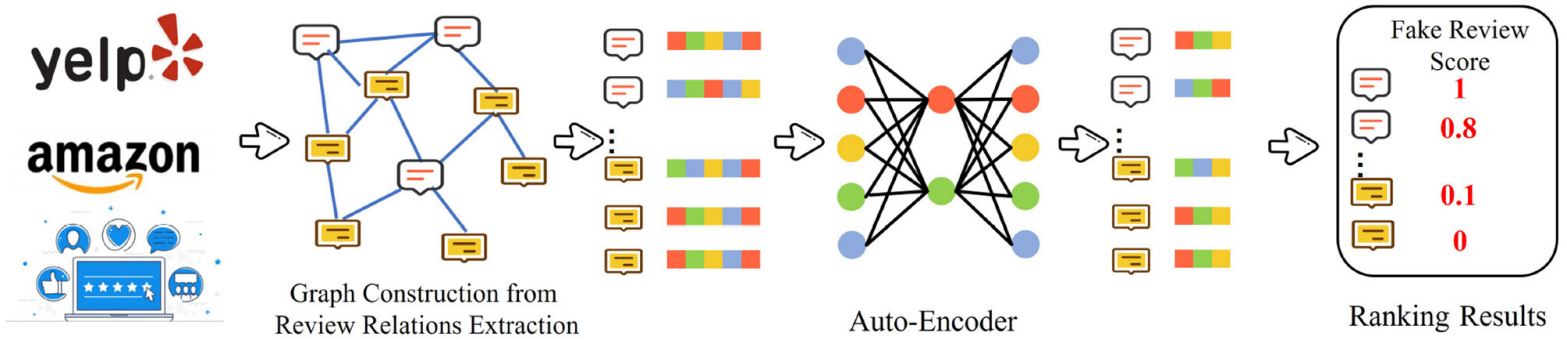
Generation-based methods with Auto-Encoder for fake review detection.

The generation-based methods aim to reconstruct and employ the input graph to serve as the supervision labels. The generation-based methods include auto-regressive, flow-based, auto-encoder methods. The detection technique for fake detection always employs the auto-encoder to learn the representation of the node. Fake review detection is formulated as a task that performs the anomaly detection tasks in attributed networks. Figure 4 illustrates a general framework of the generation-based methods with auto-encoder, which is designed for fake review detection. Wang et al. (2018) view the fake review detection problem as identifying the suspicious dense blocks in the attributed bipartite graph. They propose a deep learning model named *DeepFD* to differentiate between normal and suspicious users. DeepFD contains three primary components. The first component reconstructs the input graph by applying the encoder result of the node. The second component preserves different behaviors among diverse users. These two components preserve the structural information and behavioral characteristics. The last component detects the fake review. In the graph reconstruction component, the loss function is formulated as:


(3) $$\begin{array}{l} L_{recon}={\left\|\left(Ŝ-S\right)⊙H\right\|}_{2}^{2} \end{array}$$

wherein, ⊙ denotes the Hadamard product, Ŝ = {ŝ_1_, ŝ_2_, ……, ŝ_*i*_}, ŝ_*i*_ presents the learned graph structure of node *i*, and *S* = {*s*_1_, *s*_2_, ……, *s*_*i*_}, *s*_*i*_ denotes the initial graph structure of node *i*. *H* denotes the weight vector. By minimizing this loss function, the node representation preserves global information. In the preservation component of the user behavior, for node *i* and *j*, the distance of their embedding and the similarity are defined as follows:


(4) $$\begin{array}{l} dis_{ij}={\left\|\left({\text{h}}_{i}^{\left(K\right)}-{\text{h}}_{j}^{\left(K\right)}\right)\right\|}_{2}^{2} \end{array}$$

(5) $$\begin{array}{l} sim_{ij}=exp\left(-\lambda \cdot dis_{ij}\right) \end{array}$$

wherein, ${\text{h}}_{i}^{\left(K\right)}$ denotes the vector representations of the user node *i* of layer *K*, ${\text{h}}_{j}^{\left(K\right)}$ presents the vector representations of the user node *j* of layer *K*. The loss function of this component is formulated as follows:


(6) $$\begin{array}{l} L=L_{recon}+\alpha L_{sim}+\gamma L_{reg} \end{array}$$

wherein, $L_{recon}$ is defined in Equation 3, $L_{sim}$ denotes the *dis*_*ij*_ between all the nodes, $L_{reg}$ is L2-norm regularizer term. After obtaining embedding results, they adopt the DBSCAN algorithm, which is one of the most common density-based clustering algorithms to detect fake reviews.

Some studies adopt an auto-encoder to receive attribute and structure information for detecting anomalies. Ding et al. (2019) adopt two decoders (named structure reconstruction and attribute reconstruction decoders) to decode the result of the encoder. These encoders preserve the structure and attribute information simultaneously. According to the embedding result, this method effectively receives the anomaly score of the node. The method adopts GCN to encode the attribute network, the structure reconstruction decoder is trained by the output of the attributed network encoder *Z*, the structure reconstruction result is presented as follows:


(7) $$\begin{array}{l} \hat{\text{A}}=sigmoid\left(\text{Z}{\text{Z}}^{T}\right) \end{array}$$

The attribute reconstruction decoder utilizes the graph convolutional layer to predict the original node attributes as follows:


(8) $$\begin{array}{l} \hat{\text{X}}=f_{Relu}\left(\text{Z},\text{A}|{\text{W}}^{\left(3\right)}\right) \end{array}$$

wherein **W**^(**3**)^ denotes the learning parameter. The objective function is formulated as follows:


(9) $$\begin{array}{l} L=\left(1-\alpha \right){\left\|\text{A}-\hat{\text{A}}\right\|}_{F}^{2}+\alpha {\left\|\text{X}-\hat{\text{X}}\right\|}_{F}^{2} \end{array}$$

wherein, α denotes an important controlling parameter which balances the impacts of structure and attribute reconstructions, respectively. Finally, the anomaly score of each node *i* is calculated as follows:


(10) $$\begin{array}{l} score\left(v_{i}\right)=\left(1-\alpha \right){\left\|\text{h}-\hat{{\text{h}}_{i}}\right\|}^{2}+\alpha {\left\|{\text{x}}_{i}-\hat{{\text{x}}_{i}}\right\|}^{2} \end{array}$$

By contrast, Li Y. et al. (2019) propose a spectral convolution and deconvolution-based framework, named *SpecAE*. SpecAE encodes node attributes and topological relations at the same time. This method sharpers the features with their neighbors in order to reconstruct the features. To magnify the difference between the current node and its neighbors, the result of the encoder is formulated as follows:


(11) $$\begin{array}{l} Y=\left(1+\alpha \right)\text{X}-\alpha {\tilde{\text{D}}}^{-\frac{1}{2}}\tilde{\text{A}}{\tilde{\text{D}}}^{-\frac{1}{2}}\text{X} \end{array}$$

The propagation rule of the decoder layer is given as follows:


(12) $$\begin{array}{l} Deconv\left(\text{Z},\text{A}\right)=\sigma \left(\left(1+\alpha \right)\text{Z}-\alpha {\tilde{\text{D}}}^{-\frac{1}{2}}\tilde{\text{A}}{\tilde{\text{D}}}^{-\frac{1}{2}}\text{Z}\right){\text{W}}_{g} \end{array}$$

wherein, **W**_*g*_ denotes the trainable weight matrix in the deconvolution layer.

Generation-based methods aim to reconstruct the attribute or structure features. In these methods, the input data serve as the supervision signals. In the detection step, all methods utilize the representation of the encoder to calculate the anomaly score of the node and rank the anomalous degree.

#### 3.2.2. Contrast-Based Methods

**Figure 5:**
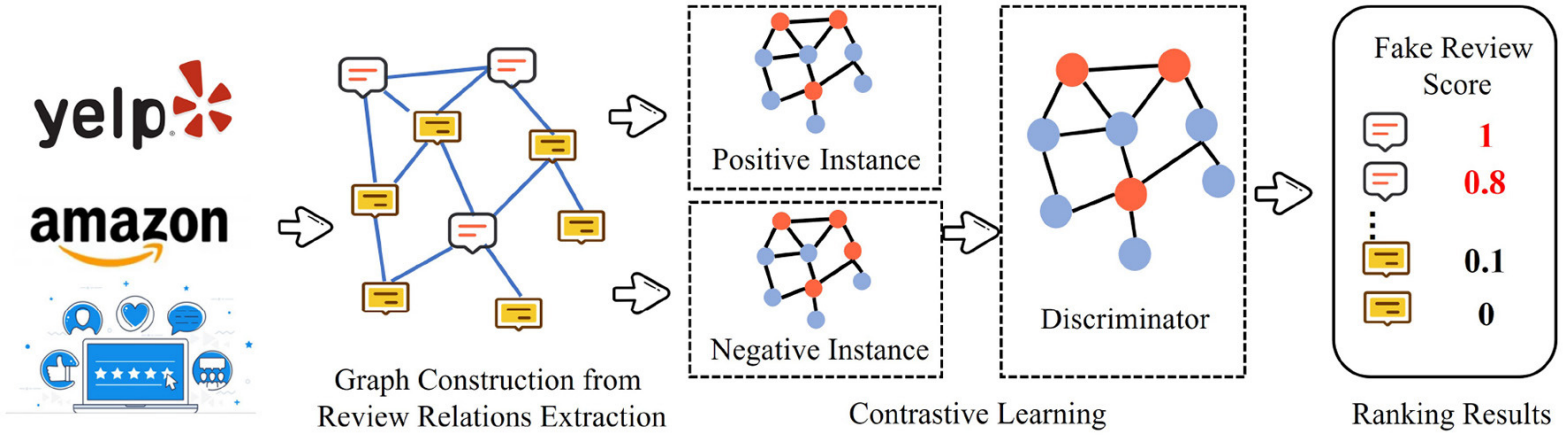
Contrast-based methods for fake review detection.

The contrast-based methods are built on the idea of mutual information maximization. These methods learn representations by contrasting positive instance pairs against negative instance pairs. Contrast-based methods are trained by a specific anomaly detection-aware target. Figure 5 illustrates a general framework of the contrast-based methods which is designed for anomaly detection.

The success of the contrast-based methods largely relies on the definition of the contrastive instance pair. The contrast-based methods that are utilized for fake review detection mainly focus on the design of instance pairs and the discernibility of positive and negative instances, respectively. Liu et al. (2021c) propose a novel contrastive self-supervised learning framework for anomaly detection on attributed networks, called *CoLA*. The instance pair in CoLA can efficiently capture local information and node attribute. Specifically, they design “target node” vs. “local subgraph”. For positive instance pairs, the initial node is set as the target node; then, the sampled subgraph is composed of the neighbor nodes of the target node. For negative instance pairs, the initial node is randomly selected from the list of nodes except the target node. Instance pairs are employed to train the GNN model for anomaly detection. The input of GNN consists of the target node, local subgraph, and label. In the GNN component, CoLA adopts GCN due to its high efficiency. Moreover, the target node embedding is the output of the GCN model, which is denoted by $z_{i}^{tn}$. The local subgraph embedding is presented as follows:


(13) $$\begin{array}{l} e_{i}^{lg}=Readout\left({\text{Z}}_{i}\right)=\overset{n_{i}}{\underset{k=1}{\sum }}\frac{{\left({\text{Z}}_{i}\right)}_{k}}{n_{i}} \end{array}$$

Secondly, in the discriminate part, CoLA applies the bilinear scoring function to produce the predicted score of a node which is calculated as follows:


(14) $$\begin{array}{l} s_{i}=Discriminator\left(z_{i}^{lg},z_{i}^{tn}\right)=\sigma \left({\text{z}}_{i}^{lg}{\text{W}}^{\left(d\right)}{{\text{z}}_{i}^{tn}}^{T}\right) \end{array}$$

wherein, **W**^(*d*)^ denotes the weight matrix of discriminator, and σ(·) presents the logistic sigmoid function. Moreover, the final anomaly score of *v*_*i*_ is obtained by computing the average value of multi-round differences between the scores of negative and positive pairs:


(15) $$\begin{array}{l} f\left(v_{i}\right)=\frac{\sum_{r=1}^{R}\left(s_{i,r}^{\left(-\right)}-s_{i,r}^{\left(+\right)}\right)}{R} \end{array}$$

wherein, *f*(·) is the mapping function of the anomaly score, which is the goal of anomaly detection. *R* denotes the number of sampling round. *S*^(+)^ and *S*^−^ are positive and negative predicted scores, respectively.

Ding et al. (2020) consider the difference between the nodes for anomaly detection. They propose adversarial graph differentiation networks (AEGIS), which learn anomaly-aware node representations to detect anomalies effectively. For graph differentiative layer *l*, the representation of the node is learned by the feature difference and node feature itself. The equation is expressed as follows:


(16) $$\begin{array}{l} h_{i}^{\left(l\right)}=\sigma \left({\text{W}}_{1}{\text{h}}_{i}^{\left(l-1\right)}+\underset{j\in N_{i}}{\sum }\alpha_{ij}{\text{W}}_{2}\Delta_{i,j}^{\left(l-1\right)}\right) \end{array}$$

wherein, $h_{i}^{l-1}$ and $h_{i}^{l-1}$ are representation of node *i* in layer *i*. $\Delta_{i,j}^{\left(l-1\right)}$ denotes the feature difference between node *i* and node *j*. After learning the anomaly-aware node representations, the second phase aims to train a generative adversarial network. This phase accurately models the distribution of normal data. The loss function, which is defined in generator *G* and discriminator *D* are as follows:


(17) $$\begin{array}{l} L_{\text{G}}={𝔼}_{\tilde{z}\sim p\left(\tilde{z}\right)}[\log(1-D(G\overset{\sim }{(}z)))] \end{array}$$

(18) $$\begin{array}{l} L_{\text{D}}=-{𝔼}_{z\sim Z}\left[\log D\left(z\right)\right]-{𝔼}_{\tilde{z}\sim p\left(\tilde{z}\right)}[\log(1-D(G\overset{\sim }{(}z)))] \end{array}$$

wherein, *z* denotes the node representation of normal node and $\tilde{z}$ denotes the generated anomaly. *G* and *D* are the generator and discriminator functions, respectively. Finally, the anomaly score of node *i* is computed as follows:


(19) $$\begin{array}{l} score\left(x_{i}^{\prime }\right)=p\left(y_{i}^{\prime }=0|z_{i}^{\prime }\right)=1-D\left(z_{i}^{\prime }\right) \end{array}$$

## 4. Datasets

The graph learning research on the fake review detection has not produced an abundant number of datastets. In this section, we provide a comprehensive review on the existing datasets which are utilised in previous studies. Table 4 presents the detailed statistics of these datasets.

**Table 4:** The statistics of fake review datasets.

**Datasets**	**#users**	**#products**	**#reviews**	**Labeled**
YelpCHI	38,063	201	67,395	Yes
YelpNYC	160,225	923	359,052	Yes
YelpZip	260,277	5,044	608,598	Yes
Amazon Reviews	34,686,770	6,643,669	2,441,053	No
Amazon FineFoods	256,059	74,258	568,454	No
Amazon Movies	889,176	253,059	7,911,684	No
BeerAdvocate	33,387	66,051	1,586,259	No
RateBeer	40,213	110,419	2,924,127	No
CellarTracker	44,268	485,179	2,025,995	No
SWMReview	966,942	15,094	1,132,373	No
Epinions	49, 290	139, 738	664, 824	No

### 4.1. Yelp

Yelp's website publishes rich crowd-sourced reviews about businesses. The Yelp dataset captures the relevant data about the businesses, reviews, and users. Specifically, the reviews in the following Yelp datasets contain various items such as product and user information, timestamp, ratings, and a plain text review.

Yelp adopts a filtering algorithm that effectively identifies fake/suspicious reviews. After the identification step, the algorithm stores the identified fake reviews into a filtered list. The filtered reviews are also made public on a business Yelp page. While the Yelp page of a business displays the recommended reviews, it is also possible to view the filtered/unrecommended reviews through a link at the bottom of the page. The Yelp anti-fraud filter is not perfect (hence the “near” ground truth); however, it has been found to produce accurate results (Weise, 2011). The following Yelp datasets are all labeled datasets that contain both recommended and filtered reviews.

#### 4.1.1. YelpCHI

YelpCHI (Mukherjee et al., 2013) is a labeled dataset that includes 67,395 reviews from 201 hotels and restaurants by 38,063 reviewers in the Chicago area.

#### 4.1.2. YelpNYC

YelpNYC (Rayana and Akoglu, 2015) is a labeled dataset that includes 359,052 reviews from 923 restaurants by 160,225 reviewers in New York City.

#### 4.1.3. YelpZip

YelpZip (Rayana and Akoglu, 2015) is a labeled dataset that includes 608,598 reviews for restaurants, starting with a zipcode number which is increased incrementally. Note that the zipcodes are organized by geography; thus, the reviews for restaurants are ordered in a continuous region of the U.S. map, including NJ, VT, CT, and PA.

### 4.2. Amazon

Amazon is a retail giant in e-commerce with billions of review data. Amazon dataset was first collected and utilized in McAuley J. and Leskovec (2013), McAuley J. J. and Leskovec (2013). To obtain this enormous data, they started with a list of 75 million asin-like strings (Amazon product identifiers) that they collected from the Internet Archive. Almost around 2.5 million of the strings had at least one review. They further divide this dataset into 26 parts based on the top-level category of each product (e.g., books, movies). The reviews in the Amazon dataset contain various items such as product and user information, ratings, and a plain text review.

#### 4.2.1. Amazon Reviews

Amazon Reviews dataset (McAuley J. and Leskovec, 2013) consists of reviews from Amazon. The dataset includes 34,686,770 reviews from 6,643,669 users on 2,441,053 products, spanning a period of 18 years from June 1995 to March 2013. Note that this dataset contains potential duplicates.

#### 4.2.2. Amazon FineFoods

Amazon FineFoods (McAuley J. J. and Leskovec, 2013) consists of reviews of fine foods from Amazon. The dataset includes 568,454 reviews from 256,059 users on 74,258 products, spanning from October 1999 to October 2012.

#### 4.2.3. Amazon Movies

Amazon Movies dataset (McAuley J. J. and Leskovec, 2013) consists of movie reviews from Amazon. The dataset includes 7,911,684 reviews from 889,176 users on 253,059 products, spanning from August 1997 to October 2012.

### 4.3. Other Datasets

#### 4.3.1. BeerAdvocate

This dataset consists of beer reviews from BeerAdvocate (McAuley J. J. et al., 2012). The data span a period of more than 10 years, from January 1998 to November 2011, including 1,586,259 reviews from 33,387 users on 66,051 beers. Each review includes ratings in terms of five aspects: appearance, aroma, palate, taste, and overall impression. Reviews include product and user information followed by these five ratings and a plain text review.

#### 4.3.2. RateBeer

This dataset consists of beer reviews from RateBeer (McAuley J. J. et al., 2012). The data span a period of more than 10 years, from April 2000 to November 2011, including 2,924,127 reviews from 40,213 users on 110,419 beers. Each review includes ratings in terms of five aspects: appearance, aroma, palate, taste, and overall impression. Reviews include product and user information followed by these five ratings and a plain text review.

#### 4.3.3. CellarTracker

This dataset consists of wine reviews from CellarTracker (McAuley J. J. and Leskovec, 2013). The data include 2,025,995 reviews from 44,268 users on 485,179 wines. Reviews include product and user information, ratings, and a plain text review.

#### 4.3.4. SWMReview

The SoftWare Marketplace (SWM) Review dataset (Akoglu et al., 2013) was collected by crawling the software product (app) reviews under the entertainment category from a popular online software marketplace. The product apps consist of a diverse set of categories (e.g., games, movies, news, sports). The complete collection includes 1,132,373 reviews from 966,842 unique users for 15,094 apps and spans 198 weeks between July 2008 and April 2012. As part of a review, a user rates a product from 1 (worst) to 5 (best).

#### 4.3.5. Epinions

Epinions (Kumar et al., 2018) is a consumers opinion site where users review items such as cars, books, movies, software, etc. In addition to the normal reviews, the consumers can assign the items numeric ratings between 1 (min) to 5 (max). Users also express their Web of Trust, i.e., a list of reviewers whose reviews and ratings have been consistently valuable. Moreover, users define Block list, i.e., a list of authors whose reviews have been consistently offensive, inaccurate, or not valuable. The dataset consists of 664,824 reviews from 49,290 users rating 139,738 different items at least once. The total number of trust statements is 487,181.

## 5. Open Issues

This section shares key challenges and open issues with respect to the search for fake review detection, including imperfect data, explainability, and lightweight models.

### 5.1. Imperfect Data

The accuracy and integrity of data are the premise to ensure the effectiveness of graph learning methods. Therefore, in fake review detection, the effectiveness of graph learning highly depends on data quality and data usability. However, the graph learning methods are often negatively affected by imperfect data (e.g., missing data, noise data, imbalanced data, and limited data). Moreover, the extensive participation of users in the review process leads to the omission of information or automatic reviews. These shortcomings cause the review data to be inaccurate and of poor quality; thus, the imperfect data leads to insufficient feature learning. As a result, graph learning outcomes will be correspondingly biased. Therefore, how to develop data-efficient fake review detection methods remains an open issue.

### 5.2. Explainability

In the practical application of fake review detection, sufficient evidence and reasons are required to indicate that a review is fake. However, commonly such explainability is the missing part of graph learning methods, which have long been criticized for their black-box nature. The graph learning-based model obtains the vector representation of the review by building the relationship between the reviews and the model feature. The detection results are then achieved through the representation. Researchers have been trying to solve the explainability problem of graph learning. However, the existing methods focus on explaining the importance of nodes or relationships in graphs, ignoring the structure factor in graph learning methods, which is more intuitive and straightforward for a human to understand. Therefore, one of the future challenges is to explore the explainability of fake review detection based on graph learning.

### 5.3. Efficiency

In the fake review detection task, the enormous size of the review data has become a significant problem (Ying et al., 2018). In such big data, the number of nodes and relationships is huge, which increases the cost of training the model. This problem is especially important in fake review detection because the model should embed nodes into low-dimensional space, detect a large number of fake reviews, and detect fake reviewers. Therefore, it is imperative to study more efficient algorithms to speed up the training and detection of large-scale data.

### 5.4. Lightweight Models

Detecting fake reviews is a comprehensive task. The enterprise is capable of detecting fake reviews by constructing complex graph structures and building graph relationships. Therefore, the detection task often performs a huge number of comparisons through a large amount of data. Furthermore, studies generally focus on how to improve the accuracy of the model by adding more parameters and layers to the models. However, fake review detection should also be performed on ordinary users' hardware or embedded platforms in real life. Since the learning models have numerous parameters and layers, utilizing them on an embedded platform has become a key challenge. To this end, the detection models should be effectively streamlined and optimized; thus, they run smoothly on devices with limited computing and hardware powers.

## 6. Conclusion

We present a survey on fake review detection methods based on graph learning in this paper. Graph-based methods are more advantageous over others because they utilize graph-structured data to formulate a binary relationship between reviews and reviewers. As a result, graph learning achieve significant performance in fake review detection. In this survey, we firstly introduce the various types of fake reviews, including untruthful opinions, exclusive reviews, non-reviews, and duplicate reviews. Moreover, we clarify different types of fake reviews by providing relevant examples for each of them. We categorize the fake review issues into three types: fake review detection, fake reviewer detection, and fake review analysis. Thirdly, we discuss the supervised and unsupervised fake review detection, which utilizes graph learning. The paper discusses the graph learning features, including the representation learning methods, detection methods, and the loss function. We analyze the unsupervised mechanisms, including generation-based and contrast-based models, respectively. Also, this paper presents a summary of the data sets that are utilized for graph-based fake review detection. Finally, we discuss the challenges and open issues, including the imperfect data, explainability, efficiency of the model, and how to propose lightweight models. This survey could be a guide for both junior and senior scholars to study the fake review issue in-depth.

## Author Contributions

SY and FX contributed to conception and design of the study. All authors contributed to manuscript writing and revision, read, and approved the submitted version.

## Funding

This work was supported in part by the National Natural Science Foundation of China under Grant 62102060.

## Conflict of Interest

The authors declare that the research was conducted in the absence of any commercial or financial relationships that could be construed as a potential conflict of interest.

## Publisher's Note

All claims expressed in this article are solely those of the authors and do not necessarily represent those of their affiliated organizations, or those of the publisher, the editors and the reviewers. Any product that may be evaluated in this article, or claim that may be made by its manufacturer, is not guaranteed or endorsed by the publisher.
